# A qualitative analysis of factors that influence Vietnamese ethnic minority women to seek maternal health care

**DOI:** 10.1186/s12884-019-2375-7

**Published:** 2019-07-12

**Authors:** Shannon McKinn, Duong Thuy Linh, Kirsty Foster, Kirsten McCaffery

**Affiliations:** 1 Sydney School of Public Health, Edward Ford Building (A27), The University of Sydney, Sydney, NSW 2008 Australia; 2 Faculty of Nursing and Midwifery, Hanoi Medical University, 1 Ton That Tung, Dong Da, Hanoi, Vietnam; 3 Office for Global Health, Sydney Medical School, Edward Ford Building (A27), The University of Sydney, Sydney, NSW 2008 Australia; 4 Kolling Institute at Northern Clinical School, Sydney Medical School, Royal North Shore Hospital, St Leonard, NSW 2065 Australia

**Keywords:** Maternal health, Service utilisation, Primary care, Vietnam, Qualitative research, Ethnic minorities

## Abstract

**Background:**

Dien Bien Province in northwest Vietnam is predominantly populated with ethnic minority groups, who experience worse maternal and child health outcomes than the general population. Various factors are associated with maternal health care utilisation in Vietnam, including ethnic minority status, which is recognised as a key determinant of inequity in health outcomes. The aim of this study is to explore how and why ethnic minority women utilise maternal health services, and the factors that influence women and families’ decisions to access or not access facility-based care.

**Methods:**

We used a qualitative approach, interviewing primary health care professionals (*n* = 22) and key informants (*n* = 2), and conducting focus groups with Thai and Hmong women (*n* = 42). A thematic analysis was performed.

**Results:**

There were three main themes. 1. Prioritising treatment over prevention: women talked about accessing health services for health problems, such as unusual signs or symptoms during pregnancy, and often saw limited utility in accessing services when they felt well, or for a normal physiological event such as childbirth. Health professionals also saw their role as being mainly treatment-oriented, rather than prevention-focused. 2. Modernisation of traditional practices: health professionals and ethnic minority women discussed recent improvements in infrastructure, services, and economic circumstances that had increased access to health services. However, these improvements were less pronounced in Hmong communities, suggesting inequity. 3. Perceptions of quality influenced service utilisation: both health professionals and ethnic minority women perceived primary care facilities to be of lower quality compared to hospital, and some women made decisions about accessing services based on these perceptions, preferring to travel further and spend more to access higher quality services. Health professionals’ perceptions of low service quality appeared to influence their referral practices, with even uncomplicated cases referred to higher level services as a matter of course.

**Conclusions:**

Primary health care facilities were technically available and accessible to ethnic minority women, however these services were likely to be underutilised if they were perceived to be of low quality. Some women had the means to access higher quality facilities, but others were limited to lower quality facilities, potentially reinforcing inequities in health outcomes.

**Electronic supplementary material:**

The online version of this article (10.1186/s12884-019-2375-7) contains supplementary material, which is available to authorized users.

## Background

The provision of adequate antenatal care and skilled attendance at delivery are widely accepted strategies for preventing infant and maternal mortality and morbidity [1–3]. Adequate antenatal care is associated with better infant survival and is an important determinant of safe delivery. While antenatal care cannot predict all potential obstetric complications, it is widely accepted that antenatal care presents opportunities to recognise and identify pregnancy risks, provide education about recognising and acting on danger signs, and monitor and support women’s health [4–6]. The risk of maternal death is highest immediately postpartum and in the following 48 h [7]. The presence of skilled birth attendants, whether a woman gives birth at home or in a health facility, is a vital intervention for preventing maternal and infant mortality [2].

There are many factors affecting the uptake of antenatal care and skilled birth attendance in low and middle-income countries (LMICs) [1, 4, 8–11]. In Vietnam, previous research has shown that factors such as belonging to an ethnic minority group, low income, low education, and living in a rural area are significantly negatively associated with maternal health care utilisation [12–14]. Ethnic minority status has been highlighted as a key structural determinant of inequity in health outcomes in Vietnam [15]. This is within a global context where ethnic and racial minorities in high-income countries as well as LMICs experience inequities in regards to health outcomes generally [16–18], and often maternal, neonatal and child health specifically [19–24].

Ethnic minority women in Vietnam are much less likely than those from the Kinh majority to attend antenatal care and to give birth with a skilled attendant present [25]. Low levels of maternal health care utilisation have also been attributed to living far from health facilities and lack of access to transport, however this overlooks the fact that utilisation of services is often low in facilities that are in close proximity to villages [26, 27]. Traditional customs and cultural differences between ethnic minority patients and health professionals are also cited. However, representations of ethnic minority practices as barriers to service utilisation can play into stereotypes that ‘other’ ethnic minorities [27]. The scapegoating of ethnic minority practices also suggests a reluctance to examine issues of satisfaction, quality, and appropriateness of health services that may contribute to low levels of service utilisation [27].

Dien Bien Province (DBP) is a mountainous province of Vietnam, predominantly populated by ethnic minority groups [28] who experience poorer health and economic outcomes than the Vietnamese average [29]. Maternal and child health outcomes in DBP are particularly poor [29–31]. Family networks play an important role in both facilitating and delaying the decision to seek maternal health care, particularly during labour, and that there are opportunities for community education around facilitators and barriers to seeking care, particularly preventive maternal care, i.e. antenatal care and skilled birth attendance/facility-based delivery for an anticipated normal birth [32]. While there has been research describing the barriers to accessing maternal healthcare services [12–14, 26, 27], there has been little research conducted that analyses how and why ethnic minority women access maternal healthcare services [27]. One previous study exploring utilisation of maternity services did not include communities located further than 3 km from a district hospital [27]. This study uses a qualitative approach to explore how and why ethnic minority women in DBP currently use and do not use maternal healthcare services, in order to illuminate the factors that influence ethnic minority women and their families in their decisions to seek and not seek maternal healthcare services, and the barriers and facilitators to preventive care seeking.

## Method

Methods for the overall study have been published elsewhere, with the study setting, recruitment, data collection and analysis processes extensively described [33, 34]. Essential information and relevant modifications are described below. Ethics approval was obtained through the University of Sydney Human Research Ethics Committee (Project No. 2015/251). The research plan was approved by the DBP Public Health Service, the Tuan Giao District Health Service, and the Vietnamese Women’s Union (VWU).

### Study design

This study utilises a qualitative, focused ethnographic design [35]. Classic ethnographies often have an open focus of investigation in which entire sociocultural fields are studied. In contrast, the focused ethnographic approach allowed us to acknowledge underlying cultural factors and social connections and behaviours, while containing our focus to a specific field of investigation (i.e. particular participant types and data collection methods) determined by pre-existing, problem-focused, and context-specific research questions [35–37].

### Study location

The study was conducted over a five week period in September and October 2015 in Tuan Giao District, DBP. Tuan Giao is a rural district with a population of approximately 82,000 people. It is 80 km away from the provincial capital, Dien Bien Phu. The study was conducted in five communes (from a possible 19), selected in cooperation with the District Health Service, and purposively sampled for a range of ethnic makeup, distances from District Hospital, and staff structure (presence of full-time doctor on staff). Commune health stations are the main providers of primary care, including antenatal care and basic maternity services. Commune level services operate under the District Health Service, which in turn operates under the Provincial Health Department. The District Hospital has surgical capacity and is the main referral point for all communes. Patients can also self-refer to district and provincial level services.

### Recruitment and participants

Twenty-two health professionals were recruited from five commune health stations, one in each of the five purposively sampled study communes. All health professionals present on the day of data collection participated with the exception of one who declined, and three who could not be interviewed due to their work duties. Forty-two ethnic minority women who were currently pregnant, or mothers or grandmothers of children under five years old, were recruited for eight focus group discussions with the assistance of the VWU. Ethnic minority women were purposively sampled for ethnicity, and level of health service engagement (women who had given birth in health facilities and women who had given birth at home). Two key informants (one village health worker, one village midwife) were recruited with the assistance of the VWU and health professionals. All participant information and consent forms were provided to participants in Vietnamese, or translated orally into local languages (Thai and Hmong) if required. All health professionals and key informants gave written consent prior to interview. Focus group participants were given a choice between a written or oral consent process. Two thirds of the focus group participants chose to give oral consent. See Table 1 for participant characteristics.

**Table 1:** Participant characteristics

Health professionals	*n* = 22	Ethnic minority women^a^	*n* = 37	Grandmothers	*n* = 5	Key informants	*n* = 2
Sex		Age (range 18–33)		Age (range 47–55)		Sex	
Male	7	< 20	7	45–49	2	Male	1
Female	15	20–24	21	50+	3	Female	1
Age (range 21–57)		25–29	5	Ethnicity		Age (range 25–27)	
< 25	1	30–34	4	Thai	5	25–29	2
25–34	10	Ethnicity		Years of school		Ethnicity	
34–44	3	Thai	28	None	1	Hmong	2
45+	8	Hmong	9	1–6	4	Years of school	
Primary health care position		Years of school		Children (n)		7–12	2
Medical assistant	11	None	5	2	1		
Midwife	6	1–6	10	3	1		
Doctor	2	7–12	19	4+	3		
Pharmacist	2	12+	3	Grandchildren (n)			
Nurse	1	Children (n)		1	1		
Years of practice (range 2 months – 38 years)		0	9	2	2		
< 10	10	1	14	3+	2		
10–19	4	2	12				
20–29	5	3+	2				
30+	3	Currently pregnant	16				

^a^Pregnant women and mothers of children under 5 years old

### Data collection

Health professional interviews were conducted in English and Vietnamese by SM, working with a Vietnamese interpreter (DTL). Focus groups were principally conducted in Vietnamese, with some interpretation into Thai and Hmong. Semi-structured interview and focus group discussion guides can be found in Additional file 1. DTL facilitated focus groups, and interpretation into ethnic minority languages was provided by local women including representatives from the VWU, People’s Committee, and a village midwife. All interviews and focus groups were audio-recorded, with the exception of one key informant interview, at the participant’s request.

### Data analysis

An independent translator translated audio-recordings and transcribed them verbatim in English. Health professional data were initially analysed using the Framework Analysis method [38, 39], with data managed in Word and Excel. Focus group data were managed using NVivo11 software. We analysed data in an iterative manner, using a hybrid coding approach that was both inductive (data-driven) and deductive (researcher-driven) to create a coding framework, which was applied to all transcripts. The data from the two main participant groups (interviews with health professionals and focus groups with ethnic minority women) each had a separate coding framework. SM then organised coded data into categories, combining the data sets at the categorical level. SM identified relationships among and between categories to generate themes. Themes were summarised and discussed with all authors.

## Results

We asked health professionals, ethnic minority women (pregnant women, mothers of children under five, grandmothers of children under five), and key informants about how and why pregnant women currently used health services, and their attitudes towards these services. The results of the thematic analysis are split into three main themes: 1. Prioritising treatment over prevention; 2. Modernisation of traditional practices, and 3. Perceptions of how quality influenced service utilisation. Themes are presented with selected supporting quotes; for more extensive supporting quotes see Additional file 2.

### Prioritising treatment over prevention

Although our discussion topics with participants were focused on maternal health care and services (Additional file 1), health professionals and ethnic minority women also discussed health service utilisation more broadly. We found that both groups (i.e. health professionals and ethnic minority women) generally talked about health care and health services in a treatment-oriented rather than a preventive sense. Perceptions pertaining to health service utilisation were often expressed in terms of responding to a current health issue, rather than preventing a future health issue.

*‘We would only go to them when we feel sick. If my health is normal, I wouldn’t go there.’* (Thai, grandmothers focus group [GFG])

Health professionals and ethnic minority women displayed several perceptions that may have a positive impact on the decision to seek health care services generally. In particular, many health professionals reported that they had made efforts in recent years to increase their engagement with communities, which had resulted in ethnic minority patients being more aware of health services, and more comfortable with approaching health services for advice and information, particularly in the context of experiencing a health problem. Both health professionals and ethnic minority women seemed to understand the role of health professionals and health services to be primarily to react to health problems, i.e. symptoms of illness and in the maternal health context, pregnancy complications. When asked about their role at the health station, health professionals often gave answers focused on examining symptomatic patients, diagnosing them, prescribing medication, and referring to the hospital.

*‘Dispensing medicine. Examining, diagnosing, providing treatment for both inpatients and outpatients. Our major job is providing primary health care, so we dispense medicine, give prescription.’* (Doctor)

In regard to maternal health, apart from the recommended number of antenatal care visits (the Vietnamese government recommends at least three visits for uncomplicated pregnancies), health professionals emphasised encouraging pregnant women to access health services when they experienced ‘unusual signs and symptoms.’ Similarly, in focus groups women spoke about the importance of going to the health station if they experienced ‘unusual’ signs, such as extreme morning sickness, irregular bleeding, and cramping. The perception that health care is primarily used when one is experiencing a health problem may influence women’s decision to access preventive care both throughout their pregnancy and in delivery. Both health professionals and ethnic minority women generally referred to antenatal care as fetal check-ups, and emphasised the importance of monitoring the health of the fetus; the importance of routine monitoring of the mother’s health was mentioned less often. Women felt less inclined to access antenatal care to monitor their own health if they felt well and did not experience obvious ‘signs and symptoms’, missing opportunities to discover hidden issues such as high blood pressure.

*‘I didn’t have any problem so I didn’t go [to the health station for antenatal care]. I never had morning sickness. Just a bit light-headed.’* (Thai, mixed focus group [MFG] of pregnant women and mothers of children under five years)

One woman described how she had just the one check-up at the District Hospital, when she was seven months pregnant with her first child. Having been assured by the doctor that there were no ‘problems’ at that time, she then delivered at her mother’s house. The same woman had visited a private clinic for check-ups at least five times during her second pregnancy due to experiencing significant nausea and lack of appetite; ‘problems’ she had not experienced in her first pregnancy. She chose the private clinic rather than the hospital because of the shorter wait times.

*‘I just wanted to check whether there’s any problem with my baby. The doctor said my baby is healthy, so I was assured until my delivery. I gave birth at home instead of the hospital.’* (Thai, MFG)

The emphasis on health problems also extended to seeking out health information; health facilities were seen by some as an inappropriate place to seek health advice if they themselves were currently healthy. Older women who wanted advice on caring for their pregnant daughters and newborn grandchildren stated that they would not ask for this kind of advice at the health station.

*‘I want to ask advice from them but I wouldn’t go there. I would just learn from others’ experience. I would only tell my children to go there when there’s any health problem.’ (Thai, GFG)*

Pregnant women and their families also had competing priorities (work obligations, caring duties, financial and time cost of travelling to health facility) to consider when making the decision to access preventive care, and saw little utility in accessing health services if they were feeling well.

*‘I only had check-up once, then no more. ( …*) *I have a lot to do. If I go, there’s no one at home to look after my child. And I have to look after the cows and buffalos too. I don’t have time for check-ups.’* (Hmong, MFG)

#### Fear as a motivating factor for accessing preventive care

Related to the theme discussed above, when women did discuss accessing preventive delivery care (i.e. a facility-based delivery), fear seemed to be an important motivating factor. While several women mentioned that they chose or planned to deliver their first child in a health facility because they were worried about their lack of experience, we also found that some women who gave birth to their first child at home experienced more worry around their second pregnancy, sometimes as a reaction to a previous complication. Others, despite having delivered at home without complications previously, indicated that they had learned health information subsequent to their first delivery, which made them more worried about a) delivering at home, and b) childbirth in general. Several women mentioned postpartum haemorrhage as a particular concern. Their previous experiences and increased health knowledge appeared to impart fear rather than confidence. These women indicated that they gave birth, or intended to give birth to their subsequent children in a health facility. While their fear seemed to motivate their decision to access preventive care prior to childbirth, they did not frame their remarks in terms of preventing a complication or emergency; rather they saw a facility-based delivery as the best way to ensure prompt availability of treatment should it be required in case of an emergency.

*‘If the baby gets out too quickly then I can’t do anything. If I’m in too much pain, I should go to the hospital. They told me that so I’m quite afraid. Many people had unexpected problems ( …*) *For example postpartum haemorrhage. I’m afraid of that. Last time I gave birth at home.’* (Thai, pregnant women focus group [PWFG])

Some family members were also worried about potential complications should their daughter or daughter-in-law give birth at home.

*‘I wouldn’t let her give birth at home. ( …*) *If something bad happens, I wouldn’t know what to do.’* (Thai, GFG)

The older women we spoke to lived in a commune close to the District Hospital, and all spoke about encouraging their daughters to deliver at the hospital, rather than at home or the health station. They also discussed encouraging hospital delivery as being a way of easing their daughters’ fears and worries about childbirth.

*‘When she’s going into labour, I would tell her not to worry because the doctors are there to help her.’* (Thai, GFG)

In contrast to those women who planned a facility delivery after a homebirth, one pregnant woman who had a negative experience (neonatal death) delivering in a health facility indicated that she intended to have her next child at home, where she previously had positive outcomes.

*‘Giving birth at home is more secure. I gave birth at the hospital and lost one child, but I gave birth to 3 children at home successfully.’* (Thai, MFG)

### Modernisation of traditional practices

Health professionals, particularly in communes with a predominantly Thai population, believed that improvements in infrastructure, health services, and people’s economic circumstances had increased the communities’ utilisation of health services. Health professionals in these communes suggested that women were able to take better care of themselves while pregnant, as they could afford to rest from heavy farm work and have better nutrition than in the past. Health professionals also commonly perceived that people in their communities valued health more highly than people in poorer communes, and believed that this drove their utilisation of health services to some extent.

*‘People here are different from other communes. They pay attention to their health and their baby’s health.’* (Doctor)

Health professionals claimed that some Thai women came monthly for antenatal care, above the minimum three that are recommended. In two communes, they no longer gave out clean birth kits because the majority of women now gave birth at a health facility. Some health professionals indicated that their communities displayed increased trust in health professionals and medical knowledge over traditional beliefs.

*‘Since the health station was established and health services were provided, people have had great trust in health staff and no longer follow superstitious rituals. They come here to get medicines and their health problems are treated so they believe in the effects of medicines.’* (Nurse)

Behaviours based on traditional beliefs and practices were often referred to by both health professionals and ethnic minority women as occurring only ‘in the past.’ However, this was sometimes contradicted within focus groups with ethnic minority women. For example some women asserted that home births were something that happened in the past, only to have another woman in the group state that she had given birth at home recently. Events and behaviours that were referred to as happening only ‘in the past’ included women doing hard farming work right up to the time of their due date, women giving birth in the fields, home births, and family members opposing the advice of health professionals.

*‘The parents would say that [oppose or contradict health professionals] in the past. It was different, they didn’t have anything to eat [laughs] but now we eat [laughs] if there is anything unusual we should go to the health station.’* (Thai, mothers of children under five years focus group [MU5FG])

Older women were particularly enthusiastic about the changes they had seen in their lifetimes as access to services and service quality improved.

*‘Nowadays it’s best to give birth at the hospital. People here don’t give birth at home anymore. In the past all pregnant women gave birth at home. All of us here gave birth at home.’* (Thai, GFG)

The situation for pregnant and postpartum women was also perceived to have improved within the family home, with older women seeing their own experiences with pregnancy and childbirth as having been ‘totally different’ and much harder than their daughters’ experiences.

*‘I tell her [my daughter] that she has it much easier than me back then, because she gets to eat everything she wants.’* (Thai, GFG).

This past hardship was particularly notable in regards to what pregnant and postpartum women were permitted to eat, with food restrictions now more relaxed. Younger Thai women in another commune reported that they do not eat certain things in the first month of the postpartum period, including buffalo, beef, and water spinach, due to fear of infections.

*‘Ten days after I gave birth, my mother let me eat chicken, but only one thigh. But that’s so luxurious already ( …*) *Things were so difficult back then. Now things are easier.’* (Thai, GFG)

#### Who is left behind?

Although antenatal care and facility-based birth had become increasingly normalised in some communities, in keeping with the narrative of modernisation, the data suggested that some people are not benefiting, or benefiting less than others, from increased access to and availability of health services. There was a marked contrast between the lowland Thai communities and the Hmong communities who live in more remote mountainous areas. Health professionals in Thai communities with a few Hmong villages reported that Hmong women presented later and less frequently for antenatal care. This was often mentioned as an afterthought to their comments about the improvements that they perceived in the rest of the commune. Behaviours that health professionals perceived as ‘harmful’ and/or relegated to ‘the past’ were also conceded by health professionals to be persistent among the poorest in their communities, both Thai and Hmong. Additionally, the improvements noted by health professionals in the three predominantly Thai communes were less remarked on by health professionals working in predominantly Hmong communes, who reported more modest achievements in improving health service utilisation and outcomes.

*‘The percentage of poor families is still high, so pregnant [women] still have to do heavy work to earn their livings, despite knowing that it’s harmful.’* (Medical Assistant and Manager of health station)

*‘And they don’t eat enough and therefore don’t have enough nutrition. We advise them to eat healthy but they can’t afford that so they don’t have enough nutrition for both mother and fetus.’* (Midwife and Manager of health station)

Data from focus groups with Hmong women and health professionals indicated a lack of available, accessible, acceptable, and affordable resources for Hmong people compared to Thai people. Hmong women reported that they were not always able to speak to a female, Hmong-speaking health professional at the health station, which made some women hesitant to access services there. Generally, the Hmong women who participated in focus groups had less Vietnamese language fluency than the Thai women, and reported difficulty and/or inability to read health information given to them by health professionals. Hmong villages were also more remote, and health stations and the District Hospital were generally less physically accessible to Hmong people. The increased distance and difficulty in reaching district level health facilities also imposed extra financial costs, as they had to travel long distances, and often required accompaniment, possibly leading to lost income for their family members.

*‘It’s harvest season now so the whole family is away at the field. She cannot bring the baby to the hospital on her own. She already called her husband. In one or two days when her husband comes home, they will go to the hospital together.’* (Hmong, MU5FG – baby had been referred to District Hospital)

### Perceptions of how quality influenced service utilisation

Those women who did access preventive health services (particularly Thai women) perceived the quality of care at primary care facilities to be poor, and were increasingly making decisions about utilising care based on these perceptions. Many of the Thai women preferred to go to the District Hospital for delivery and antenatal care as they perceived the services to be of higher quality, both in terms of facilities and personnel. Women discussed hygiene levels, space and crowding, waiting times, and staff expertise (including the fulltime presence of doctors) as quality indicators that influenced their decisions about which services to use. Private clinics were also mentioned as being a good alternative for antenatal care services for women who preferred shorter waiting times.

*‘Health staff at the health station are not as good as those at the hospital. And the facilities are not so good either, and they haven’t got as much space as the hospital.’* (Thai, PWFG)

*‘In the hospital they take my blood for testing, there are too many steps ( …*) *I want to get results quickly so I go to the private clinic.’* (Thai, MFG)

Thai women frequently mentioned the availability of ultrasound as a benefit of attending a private clinic or the hospital for antenatal care, and appeared to perceive this availability as an indication of a higher quality service. Ultrasound was not available at commune health stations.

Interestingly, health professionals, particularly in Thai communes closer to the District Hospital, also perceived a notable quality difference between the health station and the hospital, both in terms of facilities and in the abilities and confidence level of staff.

*‘They receive better care there [hospital]. They also feel safer because there are enough facilities and equipment at the hospital in case anything happens. The health staff there has more expertise too.’* (Pharmacist)

Health professionals working in a health station with inadequate physical space were especially negative about the quality of the maternal health services that they could offer.

*‘We don’t have anything. No blanket, no mosquito net, no bed. We have the heater and sterilizer though, so we can work with the tools for removing umbilical cord. We just have a table where the pregnant woman can lie on, which is very small. We only help with cases that are too urgent, otherwise we would transfer them to the hospital.’* (Medical Assistant)

Health professionals who worked in health stations compliant with the national primary care standards also criticised their facilities in comparison to the District Hospital, while stating that they had similar equipment to the District Hospital for assisting with uncomplicated deliveries, with the exception of ultrasound. Despite the apparent availability of equipment, space, and staff, health professionals at several health stations referred women to the District Hospital for anticipated uncomplicated deliveries as a matter of course. There were also suggestions that some health professionals lacked confidence in assisting deliveries, or feared complications arising that they were not equipped to detect and manage. This may have contributed to the high number of referrals. The following quotes are from two health professionals at the same health station.

*‘I’ve been thinking about how to increase the number of pregnant women giving birth here, and only transfer complicated cases to District Hospital. We don’t assist with third-child deliveries, but we should take on normal labours.’* (Medical Assistant and Manager of health station)

*‘I’m not sure whether the midwife is unconfident or she’s just avoiding the work.’* (Medical Assistant and Manager of health station)

*‘We told them that in this health centre we don’t have enough facilities and medicine, so we would recommend that they give birth at the hospital.’* (Midwife)

A village midwife also told us about an incident when two commune health stations refused to take a pregnant woman into their care because of their fear of complications they were not equipped to deal with.

*‘I called the health station because it looked like a case of premature baby. Health staff at the health station said that it might indeed be a premature birth, but they wouldn’t be able handle that case at the health station and advised me to take her to [health station in other commune]. That night I took her to [other commune] but they didn’t take the case either because they’re afraid it’s premature birth. So I took her to [hospital].’* (Village midwife)

## Discussion

These results show that ethnic minority women’s reasons for accessing and not accessing maternal health care are multifaceted and complex, with barriers beyond the physical accessibility and availability of health facilities. The main themes from our thematic analysis point towards three key findings: 1. the perceived role of health facilities generally is to provide treatment for illness or problems. This perception was found in both community members and health professionals. In the maternal health context, this perception can result in women and their families not seeing the value of accessing antenatal care and facility-based delivery. 2. Inequities exist between ethnic groups, with some communities (particularly Hmong communities) overlooked by the modernising narrative (i.e. the perception that traditional practices have been progressively and increasingly replaced and/or complemented by modern, medicalised practise), and continuing with their traditional practices. 3. Women’s and health professionals’ perceptions of quality of care in health facilities is an important factor in determining which services are utilised. Perceptions of low quality of health stations generally resulted in women either choosing or being referred by health professionals to the District Hospital to give birth, with many of those women unable or unwilling to travel making the choice to deliver at home. We do not intend to imply that delivering at home is the ‘wrong’ choice. Rather, this is an equity issue in that some women and families have the option to deliver in hospital and others do not.

The perceived role of health services as treatment-oriented rather than preventive is not unique to this population [40], and may speak to a lack of clarity about the role of health stations and health services more generally in DBP. Ethnic minority women who participated in this study generally had a non-medicalised view of pregnancy and childbirth as normal, healthy physiological states, and this was linked to their views about accessing health services. This relationship has been found to be a factor in the non-utilisation of health facilities elsewhere. Qualitative meta-syntheses of the evidence on antenatal care utilisation [4] and facility-based delivery [41] also found that women in LMICs generally viewed pregnancy as a healthy state, and so saw little reason to visit health facilities or consult health professionals during pregnancy. They also found that women resisted risk-averse approaches to maternal care and health care generally [4].

In Vietnam, a recent United Nations Population Fund (UNFPA) report on barriers to accessing maternal health and family planning services [26] found similar views among ethnic minority women across the country. The report collected data from 27 ethnic minority groups in six provinces, including the two provinces that neighbour DBP and have similar ethnic makeup and mountainous terrain. Qualitative findings from this report suggest that ethnic minority women typically access health services when they experience a complication during pregnancy, but otherwise do not find antenatal care necessary. Women who participated in the UNFPA’s qualitative study also made comments consistent with our finding that commune health stations were perceived to be primarily treatment-oriented, and associated with illness, rather than health. The UNFPA also found a belief that antenatal care and ultrasound examination could determine whether pregnancies (and subsequently labour) would be ‘easy’, indicating that further antenatal care was unnecessary, and the baby could be delivered at home, or ‘complicated’, indicating that a facility delivery was necessary [26]. We found that fear and worry were also a motivating factor for women who decided to give birth in a health facility, for both primiparous and multiparous women. The UNFPA also found that fear and lack of experience was a factor in choosing to deliver at a health facility for nulliparous women; no women in their sample had delivered at home, and then subsequently in a health facility [26].

There is evidence from countries including Sri Lanka and Malaysia that suggests that as facility-based maternity care becomes available, women tend to stop using traditional, home-based maternity care [42, 43]. Bohren and colleagues’ synthesis of the qualitative evidence on facilitators and barriers to facility-based deliveries in LMICs [41] also found the ‘desire for modernity’ [41] p.71 to be a factor driving women in some contexts towards a facility-based delivery, which they perceived as contemporary and aspirational. However, this shift has also been found to be contingent on facility-based care being accessible and of good quality; proximity and availability of services are not sufficient [26, 27]. This behavioural shift was aided by initiatives such as free transportation to health facilities and robust quality assurance measures [42]; lack of transportation and poor quality services have been found to be barriers to service utilisation among ethnic minority women in Vietnam. [26, 32, 44].

Our data found that Hmong participants, and health professionals working in predominantly Hmong communes, were less positive overall than Thai participants when speaking about the state of maternal health care and outcomes in their communities. Findings indicated a lack of available, accessible, acceptable, and affordable resources for Hmong people in DBP. In Vietnam, ethnic minority groups as a whole have much lower antenatal care attendance and skilled attendance at birth coverage than the overall national indicators and the Kinh majority [25], but there is a lack of research that disaggregates results and examines disparities between ethnic minority groups. The UNFPA found that among 27 ethnic minority groups surveyed, Hmong people were among those with the worst maternal health indicators (e.g. percentage of women attending antenatal care, percentage of births attended by skilled personnel) [26]. Research on communication between primary health care professionals and ethnic minority women in the maternal health context also indicates that Hmong women face greater communication barriers with health professionals that require culturally and contextually targeted intervention [33].

Participants (both community members and health professionals) mentioned several indicators that suggest the perception of low quality of health facilities, including long waiting times, the absence of doctors, lower level of staff expertise, no ultrasound availability, cramped facilities, absence of appropriate equipment, and lack of hygiene. We found that women who lived close to the District Hospital often preferred to attend antenatal care at the hospital, rather than the commune health station. The perception of poor quality of commune health stations, and the relative perceived high quality of the hospital was suggested as an important reason for this preference. Some women also accessed private facilities, for reasons of quality and convenience. The UNFPA also found that the main reason for the use of private facilities over commune health stations was the perceived low quality of health station services generally, with specific mention of the lack of availability of ultrasound examination, which was often used to determine whether a woman should deliver in a health facility or at home [26]. The emphasis on ultrasound examination is indicative of a wider trend in Vietnam. A study of obstetricians’ views of ultrasound use in pregnancy found that obstetricians perceived that Vietnamese women associated ultrasound with pregnancy management at the expense of other clinical examinations, resulting in missed opportunities to identify potential pregnancy complications [45]. This complements our finding that women who could choose between services often chose the District Hospital or a private facility, citing the availability of ultrasound as one of the reasons motivating their preference. Some health professionals also cited the lack of availability of ultrasound at health stations as an indicator of lower quality care, both for antenatal care and delivery services, as staff doubted their own ability to detect complications and so pre-emptively referred all labouring women to the hospital.

For many of the women in our sample, the District Hospital presented an accessible, acceptable alternative to giving birth at the commune health station. The UNFPA found that a minority of their sample delivered at a health facility, and of those the majority chose to deliver at a District Hospital [26]. However, the relative direct and indirect costs of delivering at the hospital are considerable, and out of reach of many, with an average cost of USD 130, compared to USD 20 at the commune health station, and USD 10 at home [26]. For women who are unable to access the District Hospital due to reasons of accessibility, affordability, and acceptability, their options are limited to poor quality, possibly unacceptable services at the commune health station, or home. Home births provide the psychosocial benefits of culturally appropriate family-provided care, usually without a skilled birth attendant present [26]. As such, women may perceive that there is little advantage to giving birth at the health station, compared to giving birth at home, and continue to see home as their best and/or only option [25–27].

Strengths of this study include a heterogeneous sample, involvement of health professionals and community members, a rigorous analysis process, and the involvement of local collaborators. This study had several limitations. Firstly, Vietnamese is not the first language of the ethnic minority people living in this community. All health professionals and most ethnic minority women who participated in the study spoke Vietnamese; some ethnic minority women needed to speak through local interpreters. The use of local interpreters may have resulted in some distortions in women’s responses, either self-imposed or interpreter-imposed. This is a cross-cultural study, and as such, some responses may have been misinterpreted by the authors. We have attempted to limit misinterpretations by conducting an independent translation of audio data and collaborating with a Vietnamese co-author. Any actual or potential misunderstandings were discussed by authors in regular meetings during data collection. Additionally, self-reported practice in interviews and focus group discussions may differ from actual behaviour, and there may be a related element of social desirability bias. We have tried to minimise this through use of a neutral facilitator and assuring participants about confidentiality. Finally, due to logistical and language constraints, systematic member-checking [46] and verification of our data and themes was not possible.

## Conclusions

The challenges to achieving equitable access to maternal health services are numerous and complex, and barriers exist on both the supply-side (health facilities) and demand-side (communities). However, barriers to access and improved communication between health professionals and communities are often perceived to exist mainly on the side of ethnic minority communities [27, 33], and are based on cultural stereotypes and assumptions about the cultural ‘otherness’ of ethnic minorities [27].

Health promotion approaches in DBP are often focused on increasing service utilisation by ethnic minority people, and can be didactic, one-way, and paternalistic [33, 34], perpetuating a health professional-centred approach in which health professionals tell women what they need rather than ask them what they want. This study, and others, have found that although health facilities are technically available and accessible to women, these services are likely to be underutilised if they are perceived to be of low quality [4, 26], and are not appropriately aligned with women’s social and cultural context as ethnic minority women [4, 27]. The evidence around interventions to provide culturally-appropriate maternity care is mostly from high-income countries and of low-quality [47] but it shows that culturally appropriate interventions have positive effects on the utilisation of skilled maternity care, particularly antenatal care [48]. Simple interventions, such as respecting certain preferences, e.g. accommodating traditional birthing positions and allowing relatives to be present at delivery (health facilities refusing to accommodate these practices has previously been identified as a factor that discourages ethnic minority women in Vietnam from facility-based deliveries [27]) can have a positive impact on patient satisfaction and service utilisation [49]. Interventions should be designed based on empirical data, and with the input of affected communities through participatory approaches [50]. Despite the low-quality evidence, the WHO has made a strong recommendation for providing culturally appropriate skilled care, and highlighted the need for ongoing dialogue with communities in defining culturally appropriate, high quality care that incorporates the communities cultural preferences [47].

Services will continue to be underutilised if the perceived benefits of attending a health facility are not seen by women and their families to outweigh potential harms and costs [26, 32]. Those who have the means to bypass commune level facilities may access higher quality facilities that are further away, potentially reinforcing inequities. The desirability of existing services in remote, difficult to access areas can be improved through addressing and improving the quality of local care, staff training, and communication between health professionals and communities at the primary care level [51].

## Additional files

Additional file 1: Study protocols. This Additional file contains the interview and focus group discussion guides used for data collection. (DOCX 23 kb)

Additional file 2: Table of supporting quotes. This Additional file contains a table of quotes supporting the thematic analysis findings, and extending the selected quotes included in the main body of the manuscript. (DOCX 18 kb)

## Data Availability

The datasets generated and analysed during the current study are not publicly available due to ethical reasons, but are available from the corresponding author on reasonable request.

## Abbreviations

DBP: Dien Bien Province
GFG: Grandmothers focus group
LMIC: Low and middle-income countries
MFG: Mixed focus group
MU5FG: Mothers of children under five years focus group
PWFG: Pregnant women focus group
UNFPA: United Nations Population Fund
USD: US dollar
VWU: Vietnamese Women’s Union
