# Supplementary material for: A qualitative analysis of factors that influence Vietnamese ethnic minority women to seek maternal health care
Source: BMC Pregnancy Childbirth. 2019 Jul 12;19:243. doi: 10.1186/s12884-019-2375-7 (PMC6626358; doi:10.1186/s12884-019-2375-7)
Supplement: Supplementary file 1 — Study protocols. This Additional file contains the interview and focus group discussion guides used for data collection. (DOCX 23 kb) [file 12884_2019_2375_MOESM1_ESM.docx]

# Additional file 1: Study protocols

Interview Guide – Health Professionals

**Prior to interviews in discussion with Health Station manager:**

- Number of staff
- What do they do?
- Maternal, infant and under 5 years mortality so far this year

**Introductions**

Thank you for agreeing to be interviewed. We are studying doctor-patient communication and health literacy, especially in relation to maternal health, and how your patients may understand the information that you give them about pregnancy and childbirth.

Everything you say will be strictly confidential. We will make a transcript and translation from the audio recording of this interview, but your name and identifying details will be removed.

You will be identified by a unique ID number. The interview will take around one hour. If you would prefer not to answer some questions, or would like to stop the interview at any time, that’s fine, just let me know.

Do you have any questions before we start?

| **Topics** | **Questions** | **Prompts** |
| --- | --- | --- |
| Workplace context | 1. Tell me about the community / village you work in 2. Tell me about your workplace 3. Describe a typical day at work 4. Have you had any training in maternal and infant health? | - Are you from this area? - How long have you worked here? Where did you work before? What kind of training did you do? - What kind of patients do you see? How many? - What kind of services do you provide? - What kind of training? When? Who provided the training? |
| Ante-natal care | 1. What kind of care and services does your health station provide for pregnant women? 2. What do women in your community do to take care of themselves when they’re pregnant? 3. Is there anything that pregnant women do in your village that you think is harmful? 4. Do pregnant women in this community come to the health station for check ups? 5. Why do you think pregnant women come / don’t come to the health station? 6. What are some problems that occur in pregnancy in your community? | - Is there anything that they do by themselves, without the advice of health staff? - How many of them come? Most? Not many? - Has this changed in the time you have worked here? Why? - What do you think causes these problems? |
| Childbirth | 1. Where do women in your community prefer to give birth? 2. Why do you think women in your community might give birth at home rather than the health station or a hospital? (or vice versa) 3. What kind of facilities/services does your health station provide for women who are giving birth? 4. What are some problems that occur in childbirth in your community? | - If women mainly give birth at a facility, why do you think those that still give birth at home do so? (or vice versa) - What do you think causes these problems? |
| Communication | 1. What kind of health information do you give to pregnant women and/or women who have recently had a baby. 2. Can you tell me about a time you have had a problem communicating information to a pregnant woman, or a woman with a baby/small children | - How do you give them this information? - When do you give them this information? - Do you think the women understand this information? How do you know? - What happened (i.e. was the problem resolved?) - If response is about language, can you tell me about a time you had difficulty helping a woman who spoke the same language as you understand health information? - What do you think caused the misunderstanding |
| General | 1. Is there anything frustrating about working in your community? 2. What are some good things about working in your community? 3. If you could do one thing to improve maternal and child health in this community, what would it be? |  |
| Closing | 1. Is there anything else you’d like to add? |  |

**Demographic interview schedule (turn audio recorder off)**

1. Gender: Male / Female
2. Age ____________
3. What is your position? _______________________________________________
4. What are your qualifications? _________________________________________
5. How many years have you been practicing? ­­­___________________
6. What is your ethnic group? _____________________________

**Focus Group Discussion Guide – Pregnant Women/Mothers**

**Opening remarks:** Thank you all for agreeing to take part in this focus group. We are doing a research study to find out more about the experiences of women in Dien Bien Province around pregnancy and childbirth. We are doing this because we hope that talking to you and other women about your experiences will help us find ways to improve health services in the future.

Today’s discussion will be audio-recorded. Everything you say today will be strictly confidential. Because we are talking in a group today, we also ask you to respect each other’s confidentiality, and not repeat anything you hear today outside of the group. To help us when we listen to the recording, it would be good if you could say your name before you speak. We will make a transcript and translation from the recording of this conversation, but your name and identifying details will be removed and you will be identified by a unique ID number.

This will take around two hours. If you don’t want to answer some questions, or you want to stop at any time, that’s fine, just let me know. If you do wish to leave at any time we will not be able to remove the comments you have already made from our records, because we are recording you as a group.

Do you have any questions before we start?

**Icebreakers (including both facilitators):** Introduce ourselves by name and age, number of children, stage of pregnancy.

1. **Pregnancy**

- Tell us about when you realised that you were pregnant?
  - Prompt: What happened, how did you realise, when did you realise?
- When you realised that you were pregnant, what did you do?
  - Prompt: How do you take care of yourself when you’re pregnant?
- How do you know how to take care of yourself when you’re pregnant?
  - Prompt: Who do you ask for advice?
  - Prompt: What kind of things do they tell you?
- How have you been during your pregnancy?
  - Prompt: If they mention issues / complications / illness: what did you do?
- [For mothers]: How was your pregnancy?
  - Prompt: If they mention issues / complications / illness: what did you do?

1. **Childbirth**

- If you have had a baby, can you tell us about the birth?
- Where would you like to give birth? / Where did you want to give birth?
  - Prompt: Why?
- Where did you / will you give birth?
  - Prompt: Why?
- Who was with you when you gave birth?
  - Prompt: What did they do?
- Who would you like to have with you/liked to have had with you when you gave birth?
  - Prompt: Why?
- Would you have liked anything to have been different when you gave birth?

1. **Communication and relationship with maternal health care provider**

- Do/did you visit the health station during your pregnancy?
  - Prompt: Why/why not?
- What things do/did they do there for you?
  - Prompt: What happens when you go to the health station when you’re pregnant?
  - Prompt: What are/were you looking for from the health station staff?
- Do you feel like you can ask the health worker questions about your pregnancy / childbirth / your baby?
  - Prompt: What information do they give you?
  - Prompt: Is the information helpful?

1. **Role of family and community**

- What happens/happened after you had your baby?
  - Prompt: How is your family involved with the baby? (Husband, Mother, MIL, etc).
- Does anyone (apart from a health worker) give you advice about pregnancy and having a baby?
  - Prompt: What kind of information?
  - Prompt: Is it helpful?
  - Prompt: What do you do if this advice is different from the advice that the health worker tells you.
  - Prompt: Whose advice about pregnancy and childcare do you most trust?
- Are there things that your family and or community expect you to do while you are pregnant or when your child is born?

1. **Health station**

- How far away is the health station from where you live?
  - Prompt: How do you get there?
  - Prompt: Is it difficult to get there when you are pregnant or have a small child?
- Do you know what services the health station offers for pregnant women and mothers of young children?
  - Prompt: What are they?
  - Prompt: What do you think of these services?
  - Prompt: Do you use them?

1. **Closing question**

- Is there anything else you would like to add?

**Demographic interview schedule (turn audio recorder off)**

1. How old are you? ___________ Years

2. What is your ethnic group? ________________

3. How many children do you have? ________________

4. Are you currently pregnant? ________________

5. How many years of school did you attend? ________________

6. Do you get paid for any work outside your home? ________________

6a) What do you do for work? ________________

**Focus Group Discussion Guide - Grandmothers**

**Opening remarks:** Thank you all for agreeing to take part in this focus group. We are doing a research study to find out more about the experiences of women in Dien Bien Province around pregnancy and childbirth. We know from speaking to women here and in other communes, that when they are pregnant and raising children, their mothers and mothers-in-law are one of their most important and trusted sources of information about their health, and their children’s health.

Today we would like to talk to you about what you tell your daughters and daughters in law when they come to you for advice, and about your experiences both as mothers and grandmothers. There are no right and wrong answers to the questions we will ask you this morning. We want to hear about your thoughts and opinions.

**Oral consent process – oral consent PIS and PCF**

**Icebreakers (including both facilitators)**: Introduce ourselves by name and age, tell us about your family. How many children and grandchildren do you have? Etc.

**Information and advice about pregnancy, childbirth, child health**

1. When your daughter comes to you and says that she thinks she is pregnant, what do you tell her? (or reverse – mothers might have realised daughter is pregnant before the daughter realises – what made them realise that their daughter was pregnant?)
   - What advice do you give her?
2. What do you tell your daughter about how to take care of herself while she is pregnant?
   - What do you do to care for your daughter when she is pregnant?
3. What do you tell your daughter about labour and childbirth?
4. What do you tell your daughter about taking care of her baby?
5. What information and advice does your daughter ask you for?
   - Do you ever have problems answering your daughter’s questions?
   - What do you do if you can’t answer her questions?
6. Do you receive any education from commune health staff / village health staff / Vietnamese Women’s Union about health?
   - Do you receive any education or information about maternal and child health?
   - If yes, what did you learn about?
   - Was this information helpful to you?

**Grandchildren**

1. Tell us about the birth of your grandchild(ren)
   - What happened when your daughter went into labour?
   - Where did your daughter give birth?
   - Were you there?
   - What did you do?
2. Where do you prefer your daughter to give birth?
   - Why?
3. Is there anything that your family, or the community, expects your daughter to do after she has had a baby?
4. How are you involved in caring for your daughter and her baby after she gives birth?
5. What is different now about having a baby compared to when you had your children?

**Closing question**

1. Is there anything else you would like to add?

**Demographic interview schedule (turn audio recorder off)**

1. How old are you? ___________ Years

2. What is your ethnic group? ________________

3. How many children do you have? ________________

4. How many grandchildren do you have? ________________

5. How many years of school did you attend? ________________

6. Do you get paid for any work outside your home? ________________

6a) What do you do for work? ________________
