# Supplementary material for: A qualitative analysis of factors that influence Vietnamese ethnic minority women to seek maternal health care
Source: BMC Pregnancy Childbirth. 2019 Jul 12;19:243. doi: 10.1186/s12884-019-2375-7 (PMC6626358; doi:10.1186/s12884-019-2375-7)
Supplement: Supplementary file 2 — Table of supporting quotes. This Additional file contains a table of quotes supporting the thematic analysis findings, and extending the selected quotes included in the main body of the manuscript. (DOCX 18 kb) [file 12884_2019_2375_MOESM2_ESM.docx]

# Additional file 2: Table of supporting quotes

| **Theme** | **Supporting quotes** |
| --- | --- |
| Prioritising treatment over prevention | *Health professionals:*  ‘When patients come, I examine them and give prescription.’ (Medical Assistant)  ‘People only come here when they’re sick, for example when they have a fever, sore throat, headache.’ (Medical Assistant)  ‘Dispensing medicine. Examining, diagnosing, providing treatment for both inpatients and outpatients. Our major job is providing primary health care, so we dispense medicine, give prescription.’ (Doctor)  ‘If the people’s committee is organizing a program, for example giving away blankets, then they will drop by the health station to get the medicine. Otherwise, they only come here when their health problem has become serious [laughs].’ (Doctor)  *Pregnant women/mothers of children under five:*  ‘I didn’t have any problem so I didn’t go’ (to the health station for ANC). ‘I never had morning sickness. Just a bit light-headed.’ (Thai, MFG)  ‘I just wanted to check whether there’s any problem with my baby. The doctor said my baby is healthy, so I was assured until my delivery. I gave birth at home instead of the hospital.’ (Thai, MFG)  ‘I only had check-up once, then no more. (…) I have a lot to do. If I go, there’s no one at home to look after my child. And I have to look after the cows and buffalos too. I don’t have time for check-ups.’ (Hmong, Mu5FG)  *Grandmothers of children under five:*  ‘We would only go to them when we feel sick’ (Thai, GFG)  ‘If my health is normal, I wouldn’t go there.’ (Thai, GFG)  ‘If she tells me that she feels tired or unwell, I would tell her to have check-up.’ (Thai, GFG) |
| Subtheme: Fear as a motivating factor for accessing preventive care | *Health professionals:*  ‘I think they are aware of the risks for the baby or they’re afraid of hard labour so they come here to give birth to ensure safety.’(Midwife)  *Pregnant women/mothers of children under five:*  ‘She’s worried about her baby. It’s her first baby. She doesn’t know how to take care of a newborn baby so she wants to give birth at the health station so that she can ask for advice from health staff.’ (Hmong, MFG)  ‘The second time, I knew better and thought I should go to the hospital to give birth because I was afraid. The first time I just gave birth at home.’ (Thai, Mu5FG)  ‘If the baby gets out too quickly then I can’t do anything. If I’m in too much pain, I should go to the hospital. They told me that so I’m quite afraid. Many people had unexpected problems (…) For example postpartum haemorrhage. I’m afraid of that. Last time I gave birth at home.’ (Thai, PWFG)  Facilitator: ‘Anything else you’re worried about when giving birth at home?’ Participant: ‘Too many, I can’t even begin to tell them.’ (Thai, MFG)  *Grandmothers of children under five:*  ‘I wouldn’t let her give birth at home.’ (…) ‘If something bad happens, I wouldn’t know what to do.’ (Thai, GFG)  ‘When she’s going into labour, I would tell her not to worry because the doctors are there to help her.’ (Thai, GFG) |
| Modernisation of traditional practices | *Health professionals:*  ‘In the past they didn’t have as much understanding so they would give birth at home.’ (Midwife)  ‘People here are different from other communes. They pay attention to their health and their baby’s health.’ (Doctor)  ‘We come to them, understand them and support them, so they trust us and come to us more.’ (Doctor)  ‘Since the health station was established and health services were provided, people have had great trust in health staff and no longer follow superstitious rituals. They come here to get medicines and their health problems are treated so they believe in the effects of medicines.’ (Nurse)  *Pregnant women/mothers of children under five:*  ‘The parents would say that in the past [contradict health staff advice] It was different, they didn’t have anything to eat [laughs] but now we eat [laughs] if there is anything unusual we should go to the health station’ (Thai, Mu5FG)  ‘In the past people delivered at home’ (Thai, Mu5FG)  ‘Nowadays everyone thinks we should go the health station, it’s better to trust the health staff.’ (Thai, Mu5FG)  ‘Pregnant women here have to work even when the due date is near. Some pregnant women even gave birth right on the field. But that doesn’t happen anymore, only in the past.’ (Hmong, MFG)  *Grandmothers of children under five:*  ‘Nowadays it’s best to give birth at the hospital. People here don’t give birth at home anymore. In the past all pregnant women gave birth at home. All of us here gave birth at home.’ (Thai, GFG)  ‘I tell her [my daughter] that she has it much easier than me back then, because she gets to eat everything she wants.’ (Thai, GFG)  ‘Ten days after I gave birth, my mother let me eat chicken, but only one thigh. But that’s so luxurious already (…) Things were so difficult back then. Now things are easier.’ (Thai, GFG)  ‘Now you guys eat everything (…) Back then we weren’t allowed to eat eggs or meat or anything. Just salt, dry salt [and rice], within three days of giving birth. I lost my appetite, and was very weak and didn’t have breast milk for my baby.’ (Thai, GFG)  ‘Back then there was no doctor. I gave birth at home. My mother just told me to push harder and harder. Now at the hospital they said not to push too hard. My mother told me to push really hard so that the baby could get out, but it was exhausting. Back then there were so many maternal deaths, no?’ (Thai, GFG)  *Key informants:*  ‘Now more women give birth at the health station, but that’s only because they have better knowledge.’ (Village midwife) |
| Subtheme: Who is left behind? | *Health professionals:*  ‘The percentage of poor families is still high, so pregnant [women] still have to do heavy work to earn their livings, despite knowing that it’s harmful.’ (Medical Assistant)  ‘In poor families, pregnant women don’t take good care of their personal hygiene and environmental sanitation’ (Medical Assistant)  ‘There are women who are too poor and don’t have a motorbike to go to the health centre. And they would have to pay for food and transportation to give birth at the hospital, so they prefer to give birth at home.’ (Medical Assistant)  ‘For infants, there are problems when the family is too poor, but this rarely happens now.’ (Midwife)  ‘Some women don’t know how to take care of their baby. The poorer they are, the lower level of understanding they have about how to take care of their baby. That’ll affect the health of the baby.’ (Doctor)  ‘And they don’t eat enough and therefore don’t have enough nutrition. We advise them to eat healthy but they can’t afford that do they don’t have enough nutrition for both mother and fetus.’ (Midwife)  *Pregnant women/mothers of children under five:*  ‘It’s harvest season now so the whole family is away at the field. She cannot bring the baby to the hospital on her own. She already called her husband. In one or two days when her husband comes home, they will go to the hospital together.’ (Hmong, MFG – baby had been referred to district level services by the commune health station).  *Key informants:*  ‘The financial situation is quite difficult. Some households earn enough to cover their living expenses. Other households have a lot of children and cannot earn enough to support all of them.’ (Village midwife)  ‘They understand that they should avoid heavy work and get enough nutrition. But many women can’t afford all the nutritious food. They want to eat this and that but they can’t afford it. Only those who are better off can afford that.’ (Village midwife) |
| Perceptions of how quality influenced service utilisation. | *Health professionals:*  ‘They receive better care there [hospital]. They also feel safer because there are enough facilities and equipment at the hospital in case anything happens. The health staff there has more expertise too.’ (Pharmacist)  ‘I advised them to come here to give birth but they would rather go to the hospital. They think that in dangerous situations we might not be able to transfer the women to the hospital in time. They also think that here we don’t have enough staff and medication. Even after we have advised them to come here to give birth, they still think otherwise. I told them so many times already’ (Midwife)  ‘This is the obstetrics room. This is everything we have for our obstetrics room. We don’t have tool trolleys, and no bed either because the room is too small.’ (Midwife)  ‘The health station doesn’t meet sanitation standards’ (Medical Assistant)  ‘I wish we had more facilities, so that this health station is better equipped, then we can provide better services.’ (Medical Assistant)  ‘We don’t have anything. No blanket, no mosquito net, no bed. We have the heater and sterilizer though, so we can work with the tools for removing umbilical cord. We just have a table where the pregnant woman can lie on, which is very small. We only help with cases that are too urgent, otherwise we would transfer them to the hospital.’ (Medical Assistant)  ‘I’m in a very difficult situation. We don’t have enough working space.’ (…) ‘I wish the infrastructure of this health station could be improved soon so that we have a better workplace.’ (Medical Assistant)  ‘I’ve been thinking about how to increase the number of pregnant women giving birth here, and only transfer complicated cases to district hospital. We don’t assist with third-child deliveries, but we should take on normal labours’ (Medical Assistant)  ‘I’m not sure whether the midwife is unconfident or she’s just avoiding the work’ (Medical Assistant)  ‘Here we can’t perform ultrasound scan, so we might not detect complicated cases. So I prefer to transfer them to the hospital.’ (Midwife)  ‘We told them that in this health centre we don’t have enough facilities and medicine, so we would recommend that they give birth at the hospital.’ (Midwife)  *Pregnant women/mothers of children under five:*  ‘Health staff at the health station are not as good as those at the hospital. And the facilities are not so good either, and they haven’t got as much space as the hospital.’ (Thai, PWFG)  ‘The hygiene is better in the hospital. And if there’s a problem, the Doctor will help me.’ (Thai, PWFG)  ‘In the hospital they take my blood for testing, there’re too many steps, I may have to wait until the afternoon, so I prefer the private clinic (…) I want to get results quickly so I go to the private clinic.’ (Thai, MFG)  ‘I usually went to Tuan Giao hospital for ultrasound scan to see if the fetus is healthy or not.’ (Thai, Mu5FG)  ‘I had stomach ache so I went to the hospital for ultrasound scan. They can’t do ultrasound scan at the health station.’ (Thai, PWFG)  *Grandmothers:*  ‘They (health station) don’t have enough equipment like the hospital.’ (Thai, GFG)  ‘Only give birth at the health station if there’s not enough time to go to the hospital. The health station is near. But if there’s enough time to go to the hospital, we would go there.’ (Thai, GFG)  *Key informants:*  ‘I called the health station because it looked like a case of premature baby. Health staff at the health station said that it might indeed be a premature birth, but they wouldn’t be able handle that case at the health station and advised me to take her to [health station in other commune]. That night I took her to [other commune] but they didn’t take the case either because they’re afraid it’s premature birth. So I took her to [hospital].’ (Village midwife) |
